# Unraveling the molecular basis of sensory attributes in smoking spices: a nontargeted metabolite analysis using liquid chromatography high resolution mass spectrometry

**DOI:** 10.3389/fmolb.2025.1687831

**Published:** 2025-12-05

**Authors:** Xiao Yang, Ling-Bo Ji, Xian-Kuan Huo, Ju-Fang Hao, Min Wang, Ren-Qi Wang, Bao-Jiang He

**Affiliations:** 1 Zhengzhou Tobacco Research Institute of China National Tobacco Corporation, Zhengzhou, China; 2 Henan Xinqiao Tobacco Service & Technology Co. Ltd., Zhengzhou, China; 3 Staff Development Institute of China National Tobacco Corporation, Zhengzhou, China; 4 School of Food and Biological Engineering, Shaanxi University of Science and Technology, Xi’an, China

**Keywords:** nontargeted analysis, spices, LC-MS, metabolites, flavor

## Abstract

Although natural spices are widely applied for their complex aromas and flavors, the molecular mechanisms that drive these sensory perceptions remain obscure, leaving the selection of compounds for specific taste or aromatic outcomes more art than science. In tobacco industry, this challenge has practical implications for the design and enhancements of tobacco formulations. This study employed liquid chromatography-high resolution mass spectrometry (LC-HRMS) with data-independent acquisition (DIA) to conduct a comprehensive nontargeted metabolite analysis of sensory-enhancing attributes. Fifty-seven natural spices were evaluated and categorized into three groups based on five sensory metrics obtained from smoked blank cigarette evaluations. The result showed astringency and nasal moistening scores exhibited the most significant differences. The analysis revealed 1,853 differential ion features enriched in groups of advantageous sensory perceptions. Among these, 89 metabolites were putatively identified through mass spectral matching, and 28 were confirmed using chemical standards. Sensory evaluations of artificial formulations containing these validated compounds corroborated the accuracy of the nontargeted approach in identifying flavor-enhancing metabolites. Notably, minor components were shown to play a pivotal role in enhancing sensory attributes. This study demonstrates the potential of nontargeted metabolite analysis and chemometrics as useful tools for optimizing spice formulations in the tobacco and flavor industries.

## 1 Introduction

Spices are widely utilized as flavor additives due to their distinctive aromas and associated economic and health benefits (Perez et al., 2007). Natural spices have diverse applications across industries, particularly as condiments in food and beverages. Moreover, they serve as essential ingredients in the production of personal care products, such as perfumes, cosmetics, and detergents. The unique aromatic profiles of spices have significantly influenced trade, exploration, and the development of cultures throughout history (Shiner, 2015). Various spices are mixed and formulated to enhance sensory experiences, including both smell and taste. However, the formulation of perfumes, including the selection and composition of ingredients, remains a complex and nuanced process. The growing demand for refined and sophisticated fragrances has driven the flavor and fragrance industry to adopt innovative tools. Specifically, model-based computational approaches have been developed to correlate the volume or weight ratios of selected spices with sensory ratings (Zhang et al., 2021). Despite these advances, the complex and inconsistent metabolite composition of individual spices poses challenges for accurate model predictions. To address this limitation, Santana et al. proposed using molar ratios of key flavor compounds in selected spices as input parameters for surrogate models, which demonstrated improved accuracy in predicting desirable perfume compositions (Santana et al., 2021). Consequently, identifying key flavor compounds is crucial for building robust computational models to optimize spice formulations.

Tobacco (*Nicotiana tabacum*) is one of the most widely cultivated commercial crops worldwide, particularly for recreational use. A diverse array of aromatic compounds has been extensively studied and identified as key contributors to the distinctive scent and flavor of tobacco (Wu et al., 2013). For instance, ketones, aldehydes, and oxygen-containing heterocycles provide sweet and caramel-like flavors, while nitrogen-containing compounds, such as N-heterocycles and nitriles, contribute to nutty and roasted notes (Schwanz et al., 2019). Floral and fruity aromas are attributed from compounds like geranyl linalool, damascenone, and dihydroactinidiolide (Zhu et al., 2016). Despite this diversity, the overall aroma profile of tobacco remains relatively simple, as the spectrum of flavor compounds in any given tobacco species is typically incomplete. Moreover, undesirable odorants present in tobacco result in unfavorable raw material characteristics (Banožić et al., 2020; Li et al., 2024). To overcome these challenges, the tobacco industry has made significant efforts to refine the aroma and flavor of raw tobacco materials by supplementing them with other natural spices. For instance, Hu et al. demonstrated that incorporating ingredients such as coffee, cocoa, ginger, cumin, and rhodiola significantly enhances the richness and sweetness of cigar leaves (Hu et al., 2022). Similarly, Rezk-Hanna reported improved product sweetness through the use of sweet-flavored spices derived from vanilla and fruit (Rezk-Hanna et al., 2023). Despite these advancements, the aromatic compounds in both tobacco and added plant extracts remain highly complex. Focusing on individual molecular components does not fully capture the overall aroma profile, highlighting the need for more comprehensive and advanced analytical methods to explore the full range of substances involved (Luo et al., 2013; Graves et al., 2020).

Mass spectrometry (MS)-based nontargeted metabolite analysis protocols have been widely employed to comprehensively investigate the aromatic compounds that contribute to the diverse aroma profiles of tobacco. For instance, gas chromatography-mass spectrometry (GC-MS) combined with chemometric analysis has been used to examine the relationship between the sensory qualities of flue-cured tobacco and its volatile compounds (Huang et al., 2006). Comprehensive two-dimensional gas chromatography coupled with time-of-flight mass spectrometry (GC × GC-TOFMS) has been applied to identify neutral aromatic components in tobacco (Ding et al., 2013). Gas chromatography-olfactometry-mass spectrometry (GC-O-MS) further facilitated a more focused identification of key aroma-active compounds by linking sensory characteristics to specific odorants (Song and Liu, 2018). While GC-MS is well-suited for analyzing volatile and semi-volatile compounds, it is less effective for highly polar, low-volatility, or non-volatile compounds, particularly those that are water-soluble (Choudhury et al., 2022; Baeshen et al., 2023). Additionally, the high temperatures required for GC-MS analysis can lead to the decomposition or transformation of certain water-soluble compounds, thereby limiting its applicability (Liu et al., 2013). To address these challenges, liquid chromatography-high resolution mass spectrometry (LC-HRMS) has been increasingly applied, providing a broader analytical scope for identifying key aroma precursors and neutral aromatic compounds in tobacco (Mitsui et al., 2015; Zou et al., 2023).

Unlike the hard ionization technique of electron impact (EI) used in GC-MS, which often causes extensive fragmentation, LC-HRMS employs electrospray ionization (ESI), a soft ionization method that preserves the structural integrity of fragile or large molecules for precise structural elucidation. Despite the unique strengths of LC-HRMS, the reported methods for the nontargeted metabolite analysis of natural extracts have inherent limitations. In terms of breadth, current data analysis techniques often fail to comprehensively characterize the molecular components in complex extracts, potentially overlooking critical compounds. In terms of depth, some substances are identified solely by their molecular weight, without the secondary mass spectrometry data required to determine their specific structures and properties. In LC-HRMS methods, two main MS techniques are developed for the automatic acquisition of mass spectra: Data Dependent Acquisition (DDA) and Data Independent Acquisition (DIA) (Belov et al., 2001; Collins et al., 2013). DIA enables the comprehensive collection of data by systematically fragmenting all ions within predefined, sequential m/z windows, rather than targeting specific ions for fragmentation as in DDA. This approach results in highly reproducible and more complete datasets, eliminating the bias of DDA, where only the most abundant ions are selected for fragmentation (Guo and Huan, 2020; Wang et al., 2023). However, the challenge in assigning fragments to their precursor ions in the acquired DIA datasets necessitates data deconvolution for accurate interpretation. Many algorithms and software use a peak-peak-shape matching strategy to deconvolute the DIA data, which characterizes the fragments of a precursor ion based on similarities in chromatographic retention time and peak shape (Tsugawa et al., 2015; Heuckeroth et al., 2024). Yet, in LC separations, non-selective interactions between analytes and chromatographic stationary phases (e.g., hydrogen bonding) often distort chromatographic peak shapes, complicating peak-peak-shape matching. To address this issue, we have developed the Chromatographic Retention Behavior (CRB) algorithm, which enables extraction of chromatographic peaks of real compounds from background noise even though their peak shapes are rather poor. Additionally, because the chromatographic retention behaviors of fragments follow that of their precursor ions, the mass spectra of detected precursor ions can be deconvolved (Wang et al., 2021; Wang et al., 2025). For mass spectral interpretation, the deconvolved mass spectra are then matched with mass spectral databases to attain level 2 annotations based on a dot product function (Stein and Scott, 1994; Schrimpe-Rutledge et al., 2016). These level 2 annotations are considered cost-effective but putative, as isomers may present similar mass spectra. To further validate these annotations, the chromatographic retention times of detected compounds are compared to chemical standards to determine their identities, which is referred to as level 1 annotations (Schrimpe-Rutledge et al., 2016).

In this study, LC-HRMS incorporated with DIA MS technology was applied to analyze 57 extracts of plant materials approved for use in cigarettes by the tobacco industry in China, including 24 derived from tobacco leaves and 33 from other botanical sources. Sensory evaluations were conducted to assess their flavor profiles, and the samples were classified into distinct groups using principal component analysis (PCA) based on sensory evaluation scores. A nontargeted metabolite analysis was then performed to identify metabolic differences between these groups by examining metabolites with varying abundances. This integrated analytical‒sensory evaluation framework provides a novel approach for systematically characterizing key natural metabolites associated with enhanced sensory perception.

## 2 Materials and methods

### 2.1 Sample description

The study includes 57 plant extracts, 24 from tobacco and 33 from other plants, currently utilized in the tobacco industry in China (Supplementary Data Sheet 1). The extracts were sourced from the local market by the Raw Materials Purchasing Department of the Zhengzhou Tobacco Research Institute of the China National Tobacco Corporation. They were produced by extracting corresponding plant materials with water and food-grade ethanol according to patented extraction protocols of the providers. Except the tobacco extracts, all other natural spices are permitted food-grade additives according to “National Food Safety Standards–Standards for the Use of Food Additives” (GB 2760–2024). In addition, all the natural spices used in this study also comply with the current enterprise standards of the China National Tobacco Corporation, specifically list of additives permitted for use in tobacco products (YQ 52–2024) and list of additives temporarily permitted for use in tobacco products (YQ 53–2024). The tobacco extracts represented major varieties such as Zimbabwe, Virginia, Burley, Brazil, Maryland, and American blends. The other natural spices covered a diverse range of types, including various commercial fruit extracts, tinctures, and oils.

### 2.2 Sensory evaluation

The study was approved by the Ethics Review Board of Zhengzhou Tobacco Research Institute (ZTRI ERB: 20240612T). A sensory panel consisting of six well-trained assessors (three females and three males, aged 22–30 years) evaluated the tobacco samples. The training procedures and assessor selection criteria adhered strictly to the tobacco industry standards of the People’s Republic of China (YCT 138–1998). The evaluation criteria covered five sensory dimensions: nasal sweetness, mouthful sweetness, aroma, nasal moistening, and astringency (Cui et al., 2015). Nasal sweetness referred to the perceived sweetness detected through the nasal passage, while mouthful sweetness represented the sweetness felt during inhalation. Astringency evaluated the level of dryness or puckering sensation in the mouth, and nasal moistening assessed the sensation of hydration in the nasal passage. Aroma illustrated the overall olfactory intensity of the extract. Of particular note, while the regulation of the other characteristics is outlined in the standards (YCT 138–1998), nasal moistening is a characteristic recently introduced by the tobacco industry. It refers to the comfortable sensation perceived by the nasal mucosa when smoke passes through the nasal cavity during smoking, specifically describing a soft, rounded, and comfortable feeling, as opposed to dry or pungent sensation. For this specific character, assessors were trained using a sorbitol calibration method, and the detailed training protocol is provided in the Supplementary Data Sheet 2.

The natural spices investigated are legally approved commercial products purchased from local market. Each spice was diluted to 1w% concentrations in food-grade ethanol. A 1-μL aliquot of the diluted solution was applied to blank cigarettes using an automatic sprayer commonly used for flavor addition in the tobacco industry (N800-II, BAIZE INST Co. Ltd., Zhengzhou, China). After application, the cigarettes were equilibrated in a chamber maintained at a consistent temperature (i.e., 22 °C ± 2 °C) and humidity (i.e., 60% ± 5%) for 1 week. The uniformity of flavor distribution along the longitudinal axis of the treated cigarettes has been confirmed through verification in both laboratory experiments and industrial-scale production. The evaluation was performed in a controlled sensory testing environment to minimize external influences on perception. A trained panel of reviewers conducted the sensory evaluation using a blind assessment method to ensure objectivity and accuracy. Each extract was impregnated in 6 pieces of blank cigarettes for evaluation by each assessor. The extracts were assessed in a randomized order. Between the assessments of two extracts, the assessor was treated with clean water and rested for 10 min to cleanse their palates. During the sensory evaluation progress, assessors independently recorded their scores to avoid bias, and the data were subsequently analyzed to determine sensory differences between extracts and identify the attributes contributing most significantly to the overall sensory experience. The scoring system allocated 0–50 points for nasal sweetness, mouthful sweetness, nasal moistening, and astringency, and 0–100 points for aroma. A score of 0 indicated the absence of the sensory characteristic, while scores of 50 (or 100 for aroma) represented the strongest sensory expression. This standardized methodology ensured a rigorous and consistent evaluation of the sensory characteristics of the natural extracts.

### 2.3 Nontargeted metabolite analysis

The samples were stored in 10-mL amber vials at room temperature. For liquid samples, 0.1 mL of the raw liquid was transferred into a labeled centrifuge tube with a pipette, followed by the addition of 0.9 mL methanol for dilution. The mixture was vortexed for 15 min. For solid samples, approximately 10 mg of the sample was weighed into a labeled centrifuge tube, and 1 mL of methanol was added. The sample was then subjected to ultrasonic treatment for 15 min. After thorough mixing, 0.1 mL of the resulting dilution was pipetted into a new centrifuge tube, followed by the addition of 0.9 mL methanol to achieve a second dilution, resulting in a 1000-fold dilution. The mixture was centrifuged at 10,823 g for 15 min at 4 °C on a centrifuge (TGL-16M, Cence Co. Ltd., Changsha, China) to remove insoluble residues. The supernatant was then transferred to HPLC vials for subsequent LC-HRMS analysis.

Nontargeted metabolite analysis was performed using a Waters UPLC system coupled with a Sciex TripleTOF 5600™ MS instrument (Framingham, United States). Chromatographic separations were achieved using nine-gradient reversed-phase liquid chromatography (RPLC) and hydrophilic interaction chromatography (HILIC) systems. The RPLC separation was performed on a Waters ACQUITY UPLC HSS T3 Column (130 Å, 1.7 µm, 2.1 × 100 mm), and the HILIC separation utilized a Waters ACQUITY UPLC BEH Amide Column (130 Å, 1.7 µm, 2.1 × 100 mm). Binary mobile phase consisting of water and acetonitrile was used. For RPLC, both the two phases contained 0.1% formic acid. In HILIC mode, the water phase was supplemented with 5 mM ammonium acetate. Flow rate of the mobile phase was set as 0.3 mL/min and oven temperature was 40 °C.

**FIGURE 1:**
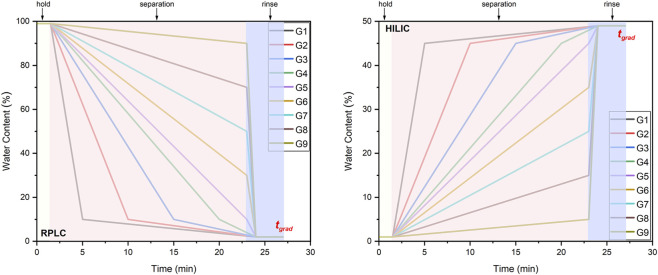
Systematic nine-gradient liquid chromatographic (LC) separation methods designed for reversed-phase liquid chromatography (RPLC) and hydrophilic interaction liquid chromatography (HILIC) modes.

A standardized data independent acquisition (DIA) mass spectrometry (MS) method and data deconvolution algorithm were described in detail in our previous reports (Wang et al., 2021; Wang et al., 2023). In brief, TOF MS datasets were acquired in the range of 70–1,200 Da, and dwell time was set as 150 ms. The DIA method employed 12 variable SWATH windows, and each MS/MS scan costed 50 ms. Analysis data in both positive and negative ion modes were acquired. The declustering potential (DP), collision energy (CE), and collision energy spread (CES) were set as 80 V, 35 V, and 15 V, respectively. Samples were mixed at equal volume to make a quality control (QC) sample. The QC sample was analyzed with nine altered LC gradients (Figure 1) and both precursor ion peaks and MS/MS spectra of nontargeted metabolites were deconvolved by the chromatographic retention behavior (CRB) algorithm (Wang et al., 2021; Wang et al., 2023; Wang et al., 2025). Then, each sample was analyzed with the 5-th LC gradient (G5). Nontargeted ion peaks in the LC-MS/MS data of each specified sample were searched in a targeted mode based on the list of nontargeted ion features summarized by CRB from the datasets of QC sample. The samples were analyzed in both HILIC and RPLC. As the samples were analyzed in both positive and negative ion modes, four DIA datasets (i.e., positive-RPLC, negative-RPLC, positive-HILIC, and negative-HILIC) were acquired for other samples.

### 2.4 Data processing and statistical analysis

Raw data from the nontargeted metabolite analysis were processed using the CRB algorithm to deconvolute ion features (Wang et al., 2021). The resulting dataset included the m/z, retention time, and mass spectrum for each deconvolved ion feature. Subsequently, the peak area of each ion feature was integrated across all investigated samples. The data were then submitted to the online platform Metware (https://cloud.metware.cn/#/home) for principal component analysis (PCA) to reduce data dimensions for sample grouping. For the comparison of two spice groups with differing sensory perceptions, ion features exhibiting a significantly higher (i.e., fold change > 2-fold and p value <0.05 in Welch’s t-test) peak area in the group with relatively higher sensory scores were identified. The extracted ion chromatograms (XICs) of these features were summed to generate the abundant ion chromatograms (AICs). As such, the AICs illustrate the presence of abundant ion features associated with enhanced sensory perception. In comparison, the data sets collected with the 5^th^ gradient (G5, Figure 1) were also deconvolved by MS-DIAL (v5.5.251021-net48) for comparison. The software parameters were set as default with minor adjustments. Specifically, feature detection parameters include: smoothing method: linear weighted moving average; smoothing level: 3; minimum peak height: 1,000; minimum peak width: 5 s; average peak width: 30 s; mass slice width: 0.01; retention time begin: 2 min; retention time end: 27 min; MS1 mass range begin: 70 Da; MS1 mass range end: 1,200 Da; MS2 mass range begin: 50 Da; MS2 mass range end: 1,200; MS1 tolerance for centroid: 0.01 Da; MS2 tolerance for centroid: 0.025 Da; accuracy type: is accurate; max charge number: 2; considering Br and Cl for isotopes: false; max isotopes detected in ms1 spectrum: 2. DIA data deconvolution parameters were set as: sigma window value: 0.5; amplitude cut off: 0; keep isotope range: 5; exclude after precursor: true; keep original precursor isotopes: false; is do andromeda ms2 deconvolution: false; andromeda delta: 100; andromeda max peaks: 12; target CE: 0.

### 2.5 Annotation

Level 2 annotations of nontargeted metabolite analysis ion features were attained by matching their experimental mass spectra with MassBank of North America (MoNA) (https://mona.fiehnlab.ucdavis.edu/), with mass error (ME) less than 10 ppm and dot product (DP) value beyond 0.4 (Schrimpe-Rutledge et al., 2016). The level 2 annotations were further verified with chemical standards to achieve level 1 identification if their chromatographic retention time difference is within 0.1 min.

### 2.6 Validation and quantification

For validation, all available chemical standards were accurately weighed (1 mg each) and transferred into labeled microcentrifuge tubes. Subsequently, 1 mL of solvent (e.g., methanol, ethanol, or water, dependent on the solubility) was added to each tube to dissolve the standard. The solutions were vortexed for 2 min and subjected to ultrasonic treatment for 10 min to ensure complete dissolution. Afterward, 100 μL of each solution was transferred to a new microcentrifuge tube and diluted with 900 μL of the same solvent. The diluted samples were then centrifuged at 10,823 g for 15 min at 4 °C. Finally, 500 μL of the supernatant was carefully collected and transferred to HPLC vials for analysis.

The quantification of identified compounds in the spice extract was performed using a single-point standard addition method to correct for matrix effects. To do so, aliquots of the spice extract were spiked with a known concentration of the target analyte standard, ensuring that the concentration of the spiked standard was of the same magnitude as the analyte concentration in the sample. Both spiked and unspiked samples were analyzed using the same LC-MS method under identical conditions. The ratio of the peak area of the spiked analyte to that of the unspiked analyte in the sample was calculated. The concentration of the analyte in the original sample was determined using the following Equation 1:

$$C_{s}=\frac{C_{std}\times {PeakArea}_{sample}}{{PeakArea}_{spiked}-{PeakArea}_{sample}}$$ (1)

Therein, *C*_*s*_ is the concentration of the compound in the sample. *C*_*std*_ is the concentration of the spiked standard. *PeakArea*_*sample*_ and *PeakArea*_*spiked*_ are peak areas of the compound in unspiked and spiked samples.

## 3 Results and discussion

### 3.1 Sensory scores of tobaccos and potential supplementary natural spices

**FIGURE 2:**
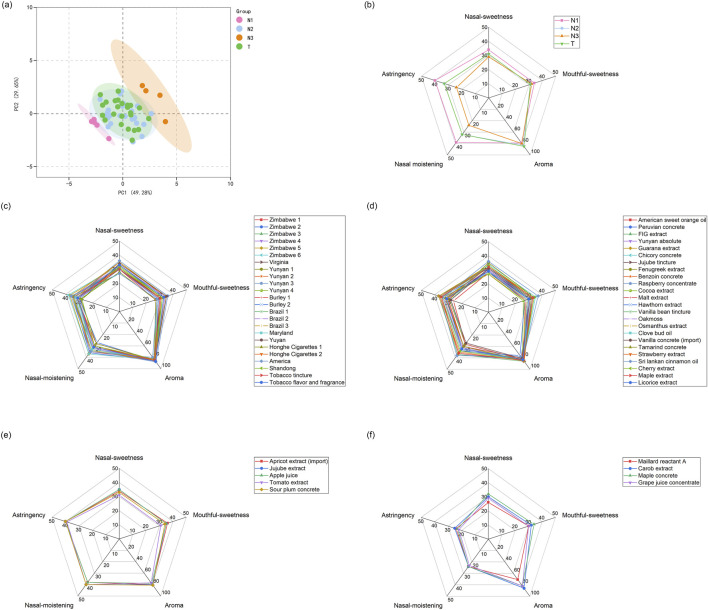
Sensory evaluation of the investigated spices: **(a)** Principal Component Analysis (PCA) illustrating the classification of spices; **(b)** Radar diagram showing the averaged sensory scores for each spice group; **(c–f)** Radar diagrams depicting the sensory scores for spices in the T, N2, N1, and N3 groups, respectively. T represents the group of tobacco extracts, and N1, N2, and N3 are natural extracts from different plants but classified according to the PCA analysis result of their sensory scores.

The natural spices were evaluated by 6 experts, and the scores given showed high consistency. Only 3% of the scores were identified as outliers, falling outside the established bounds based on the interquartile range (IQR) method. The average standard deviation of the sensory metrics across all the samples was marginally low at 1.45% (Supplementary Data Sheet 1). The 57 assessed samples were broadly categorized into two groups: 24 tobacco extracts (T group) and 33 natural extracts from different plant materials (N group). PCA of sensory evaluation scores revealed that 24 natural extracts shared similar sensory characteristics with tobacco, while 9 exhibited distinct differences. Based on the PCA results, the natural extracts were further divided into three subgroups: N1, N2, and N3, highlighting the diverse sensory profiles of natural spices compared to tobacco extracts (Figure 2a).

Radar diagrams reveal that tobacco extracts were characterized by a strong aroma (average score: 84.9) but exhibited moderate levels of nasal sweetness (31.3), mouthful sweetness (31.3), astringency (32.3), and nasal moistening (32.6). These findings suggest that enhancing these sensory parameters might require supplementation with natural spices. The sensory profiles of N2 group spices closely aligned with those of tobacco extracts as shown in Figure 2b. In another word, the scores of N2 extracts showed marginal advantages over the tobacco extracts in T group, within the sensory evaluation framework. As such, N2 extracts as additives were unlikely to significantly improve the sensory evaluation scores of cigarettes. On the other hand, despite their limited impact on parameters such as nasal sweetness, mouthful sweetness, nasal moistening, and astringency, the similarity of N2 extracts to tobacco suggested that they could introduce unique plant-derived aromas without negatively affecting the overall sensory profile (Figures 2c,d).

In contrast, significant differences emerged with the N1 and N3 groups, particularly in astringency and nasal moistening scores (Figure 2b). The N1 group, consisting of apricot extract, sour plum concrete, jujube extract, apple juice, and tomato extract, showed higher scores for astringency (average score: 33.6) and nasal moistening (average score: 34.2) compared to tobacco extracts. These findings suggest that N1 extracts might be able to enhance these sensory parameters in tobacco products, offering an advantage over the moderate scores observed in tobacco extracts. This group demonstrated a relatively consistent range of scores across sensory parameters, including nasal sweetness (31–35), mouthful sweetness (31–36), aroma (77–81), nasal moistening (38–40), and astringency (39–40), highlighting their potential for balancing and improving sensory profiles when combined with tobacco (Figure 2e). On the other hand, the N3 group, consisting of Maillard reactant A, carob extract, maple concrete, and grape juice concentrate, exhibited lower scores for astringency (average score: 23.8) and nasal moistening (average score: 23.5) compared to tobacco extracts. According to the sensory evaluation results, these extracts were not favorable as Supplementary Material for sensory enhancement in tobacco products. The group showed a broader range of scores across sensory parameters, including nasal sweetness (26–32), mouthful sweetness (30–34), aroma (71–86), nasal moistening (23–24), and astringency (22–25). The lower scores in key sensory parameters suggest that N3 extracts were unlikely to contribute significantly to improving the overall sensory profile (Figure 2f).

The distinct sensory profiles of the N1 and N3 groups, particularly in terms of astringency and nasal moistening, highlight their unique characteristics compared to tobacco extracts. N1 group extracts, when used as supplements to tobacco formulas, showed potential for formulations requiring balanced sweetness and moistening effects. In contrast, N3 group spices, although offering a broader range of aromas, consistently displayed lower astringency and nasal moistening scores, which might limit their suitability as formulation ingredients in the tobacco industry. This classification provided valuable insights into the potential applications of natural extracts, emphasizing the nuanced sensory contributions each group can offer in various contexts.

### 3.2 Evaluation of compositional variations between sample groups through nontargeted metabolite analysis

**FIGURE 3:**
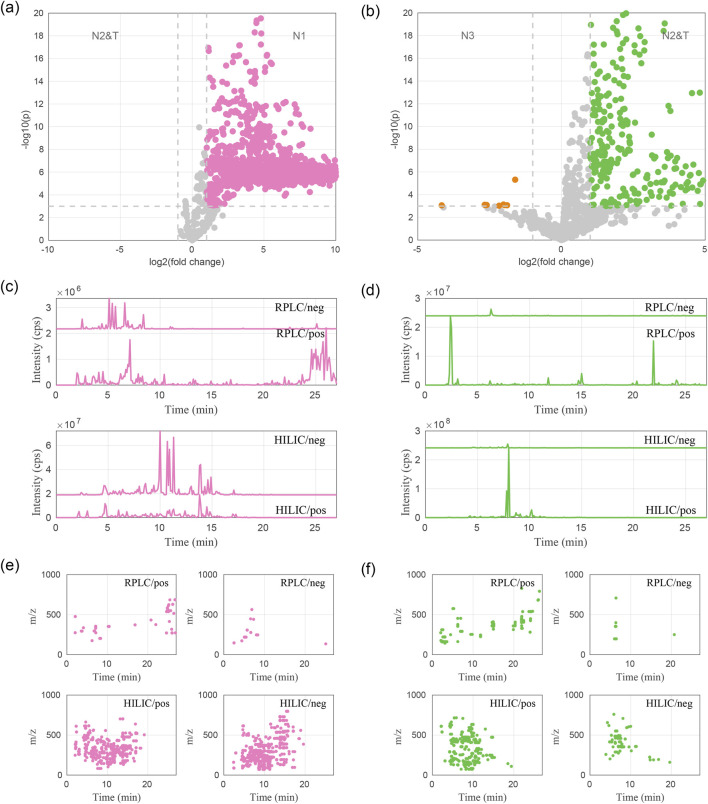
Differential ion features identified through statistical analysis between sample groups: **(a,b)** Volcano plots highlighting differential ion features; **(c,d)** Accumulated ion chromatograms of differential ion features showing higher abundances in the N1 group and N2&T group, respectively; **(e,f)** Distribution of differential ion features based on m/z and retention time for the N1 group and N2&T group, respectively.

A statistical analysis was conducted to identify differential ion features using thresholds of fold change >2 and p < 0.05. Since the natural extracts in the N2 group exhibited sensory evaluation scores similar to those of the tobacco extracts (T group), these two groups were combined into a single group (N2&T) for the analysis. Comparisons were then made between the N1 group and the N2&T group, as well as between the N2&T group and the N3 group, to identify ion features associated with the observed differences in sensory evaluations. The analysis revealed that the N1 group contained 1,250 ion features significantly more abundant than those in the N2&T group, as indicated by the pink spots in Figure 3a. Conversely, the N2&T group exhibited 603 ion features with higher abundances compared to the N3 group, represented by the green spots in Figure 3b. These 1,853 differential ion features were linked to metabolites associated with enhanced sensory attributes. In comparison, MS-DIAL was used to extract ion features for the analysis of differential ion features. A total of 975 ion features were identified as more abundant in the N1 group compared to the N2&T group, while 320 ion features were found to be more abundant in the N2&T group than in the N3 group. Both data deconvolution methods revealed an overlap of 851 abundant ion features (AIFs) between the N1 and N2&T groups, and 232 AIFs between the N2&T and N3 groups (Supplementary Data Sheet 3).

To better visualize the distribution of these relatively more abundant ion features (AIFs, i.e., fold change > 2-fold and p value <0.05) across the sample groups, abundant ion chromatograms (AICs) were constructed for each group (Figures 3c,d). The AIC provided a two-dimensional representation of retention time and intensity, offering a clear view of the separation and abundance of AIFs in reverse-phase liquid chromatography (RPLC) and hydrophilic interaction liquid chromatography (HILIC) modes. The AIC for the N1 group (Figure 3c) indicated that most AIFs exhibited low hydrophobicity, with retention times under 10 min in RPLC mode. In HILIC mode, these features were well-distributed across a retention time range of 5–15 min. Additionally, hydrophilic compounds in the N1 group predominantly exhibited higher intensities in negative ion mode, suggesting their acidic nature. In contrast, the AIFs in the N2&T group (Figure 3d) were less diverse and were confined to specific retention time windows in both RPLC and HILIC modes. These AIFs likely corresponded to compounds with higher hydrophobicity, characterized by retention times exceeding 20 min in RPLC mode and under 10 min in HILIC mode. Furthermore, these features exhibited greater intensities in positive ion mode, indicating they might include nitrogenous compounds such as amines, pyridines, or pyrimidines (Schumacher et al., 1977).

AIFs associated with enhanced sensory perception were predominantly small molecules with masses under 500 Da (Figures 3e,f). These metabolites were better resolved in HILIC mode, where more AIFs were detected, suggesting that these compounds likely contained hydrophilic moieties.

### 3.3 Annotated metabolites responsible for improved sensory perceptions

**FIGURE 4:**
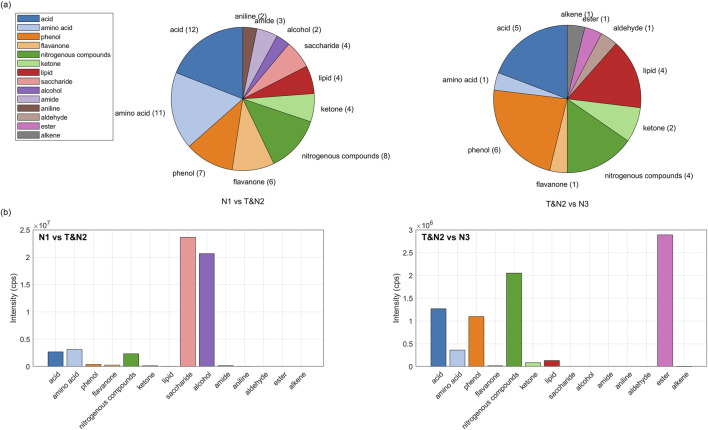
Distribution of level-2 annotated flavor compounds across various chemical categories: **(a)** Pie charts displaying the number and proportion of annotated compounds in each category; **(b)** Bar charts representing the accumulated intensities of annotated compounds within each chemical category.

The sensory differences among the N1, N3, and T groups were primarily attributed to nasal moistening and astringency, with abundant ion features (AIFs) providing molecular-level insights. From the 1,250 AIFs identified in the N1 group, 63 level 2 annotations were identified, while 26 Level 2 annotations were derived from the 603 AIFs in the N2&T group. These annotated metabolites were categorized into 14 classes (Supplementary Data Sheet 4). A comparative analysis revealed that acids, amino acids, phenols, flavanones, nitrogenous compounds, ketones, and lipids were the predominant flavor compounds shared by both the N1 group and the N2&T group. However, the N1 group uniquely featured additional flavor compounds, such as anilines, amides, alcohols, and saccharides, while the N2&T group was distinguished by unique compounds, including alkenes, esters, and aldehydes (Figure 4).

Saccharides and alcohols, such as xylose (fold change 31, p = 3.7 × 10^−3^), glucose (fold change 31, p = 2.1 × 10^−3^), mannitol (fold change 78, p = 7.7 × 10^−4^), 1,6-anhydro-glucose (fold change 8, p = 4.3 × 10^−3^), and isomaltose (fold change 11, p = 2.4 × 10^−3^), were identified as key contributors to the improved nasal moistening perceptions of the N1 group. These compounds play a critical role in nasal moistening, supported by studies showing that during tobacco processing, starch degradation releases considerable amounts of maltose and glucose (Yamaguchi et al., 2013). However, reducing sugars in tobacco can be further oxidized or transformed through Maillard reactions or caramelization, leading to reduced sugar content in the final product (Banožić et al., 2020). Given the positive correlation between sugar content and sensory evaluation scores of tobacco products (Chen et al., 2021), manufacturers often supplement sugars to neutralize the harsh taste and throat impact of tobacco smoke, while enhancing sweetness and the pleasant caramelized aroma (Talhout et al., 2006). Mannitol, with its hygroscopic properties, likely enhances moisture retention and texture, contributing to favorable sensory attributes (Balbas et al., 2015). Therefore, it probably contributes to the superior nasal moistening scores of the N1 group by improving moisture retention and texture.

Organic acids, due to their hygroscopicity and sourness, also play an essential role in improving nasal moistening and astringency (Sowalsky and Noble, 1998; Jing et al., 2018). The N1 group contained a higher diversity of acids, with 12 unique acids compared to only 5 in the N2&T group. Notably, quinic acid (fold change 188, p = 2.5 × 10^−3^) and galacturonic acid (fold change 175, p = 3.3 × 10^−3^) were key contributors. Quinic acid, a well-characterized flavor enhancer found in plant materials such as coffee, tea, and tobacco leaves, imparts a characteristic astringency and modulates sensory profiles during heating through reactions with anhydrides and other organic acids (Deshpande et al., 2016; Gigl et al., 2021; Yeager et al., 2023). Additionally, quinic acid contributes to phenolic yields during pyrolysis, further enhancing sensory attributes (Wang et al., 2013). D-galacturonic acid is involved in galactose metabolism of plants, playing a vital role in the growth and development of plants, and affecting the quality of flavor (Chen et al., 2023). D-galacturonic acid is also an enzymatic catalyzed degradation product of covalently-bound pectin, during post-harvest ripening of fruits. Thus, the contents of D-galacturonic acid increase while nanostructure of plants changes during post-harvest ripening and drying stress. Such nanostructural changes is favorable for the release of flavor compounds (Ni et al., 2023).

Free amino acids also differentiated the N1 group. The N1 group contained 11 free amino acids, including alanine, aspartic acid, asparagine, and maleamic acid, whereas the N2&T group exhibited only one free amino acid, pyroglutamic acid. The amino acids in the N1 group were relatively more hydrophilic, with retention times of 13.97–14.31 min compared to 5.19 min for pyroglutamic acid in the T group. This indicates that tobacco extracts lacked polar amino acids. Although free amino acids were not considered the major contributors, they did contribute to astringency of foods (Vrzal et al., 2021; Zhou et al., 2022). As such, the less advantageous sensory perception of N2&T group, such as nasal moistening and astringency by smoking, could be associated with its lack of hydrophilic amino acids. Accordingly, the supplementary of hydrophilic free amino acids into samples of N2&T group could probably enhance their sensory perceptions.

In contrast, the N2&T group showed significantly higher abundances of esters, such as acetyl tributyl citrate (fold change 208, p = 1.6 × 10^−5^), and nitrogenous compounds, including 6-methyl isoquinoline (fold change 3, p = 7.5 × 10^−4^) and 2-methyl indole (fold change 194, p = 0.045). These compounds are known to enhance the characteristic sensory ratings of tobacco leaves, with isoquinoline and indole derivatives contributing significantly to the flavor (Wu et al., 2022; Zhao et al., 2023). This group also contained distinctive flavor compounds such as proline betaine (fold change 64, p = 1.7 × 10^−5^) and nicotinic acid (fold change 40, p = 0.04), which are likely biosynthetic precursors of nicotine in tobacco leaves (Cordell, 2013).

### 3.4 Validation with chemical standards and formulation design

Sensory evaluation highlighted significant differences among the sample groups in terms of astringency and nasal moistening. The nontargeted analytical approach was applied to investigate differential ion features associated with unknown molecular components that showed positive correlations with enhanced sensory evaluation outcomes. From a total of 89 putatively annotated metabolites linked to these ion features, validation with available chemical standards identified 28 nonvolatile compounds (31.5%). Of these, 23 compounds were more abundant in the N1 group compared to the N2&T group, while 5 compounds were more abundant in the N2&T group compared to the N3 group (Supplementary Data Sheet 5). The quantities of each identified compounds in 57 samples were measured through standard addition method (Table 1 and Supplementary Data Sheet 5). Notably, three major compounds, glucose, isomaltose, and mannitol, were present in the N1 group at average concentrations of 8912.86 ppm, 3466.33 ppm, and 1,169.64 ppm, respectively. These compounds are well-known for their applications in tobacco products. In contrast, the concentrations of other compounds were significantly lower, ranging from 629.33 ppm for quinic acid to 0.05 ppm for hispidulin 4-O-β-D-glucopyranoside.

**TABLE 1:** Concentration of validated compounds in advantageous spice groups.

Name	Compared groups	Average concentration (ppm)			
		N1	N2	N3	T
Vitamin C	N1 vs. N2&T	7.79	0.89	0.53	0.15
Quinic acid		629.33	7.01	85.52	3.54
3,5-Dimethoxycinnamic acid		2.28	0.21	0.67	0.66
Galacturonic acid		28.80	0.59	0.00	0.15
N-fructosyl isoleucine		14.82	1.15	1.34	0.11
N-acetyl-aspartic acid		0.86	0.14	0.55	0.05
Gamma-aminobutyric acid		135.69	1.19	4.81	0.71
Alanine		292.80	0.25	0.11	0.53
Muramic acid		48.90	1.99	10.66	0.60
Asimilobine		1.65	0.06	0.17	0.02
Hispidulin 4-O-beta-glucopyranoside		0.05	0.01	0.06	0.00
Procyanidin B2		0.20	0.11	0.15	0.00
Vitexin		32.74	21.82	52.09	0.58
Niacinamide		6.10	0.25	0.27	0.22
Fusaric acid		0.34	0.01	0.01	0.01
Mannitol		1,169.64	23.99	304.66	5.89
Adenosine		76.43	0.91	6.64	0.39
N-acetylcytisine		0.21	0.09	0.03	0.01
Cytidine		56.69	2.87	12.52	1.69
2-Deoxyuridine		2.21	0.25	0.42	0.10
Glucose		8912.86	452.90	126.11	118.93
1,6-Anhydro-glucose		358.64	61.10	123.33	30.91
Isomaltose		3466.33	526.00	182.77	102.34
Proline betaine	N2&T vs. N3	0.14	2.51	0.31	11.43
4-Caffeoylquinic acid		14.55	9.91	14.23	151.22
Feruloyltyramine		2.76	5.18	8.00	128.63
7,8-Dihydroxycoumarin		0.88	3.48	1.42	6.76
Acetyl tributyl citrate		276.82	478.48	146.16	482.61

**FIGURE 5:**
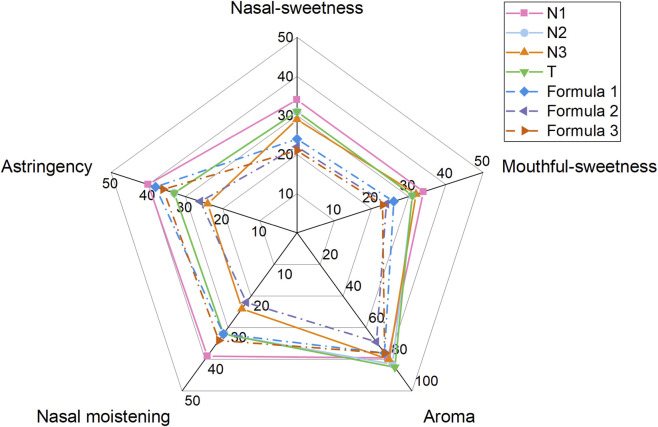
Radar diagrams illustrating the sensory evaluation scores for natural spices and artificial formulas designed based on nontargeted metabolite analysis results. T represents the group of tobacco extracts. N1, N2, and N3 are natural extracts from different plants but classified according to the PCA analysis result of their sensory scores. Formula 1 is composed of all 28 validated metabolites matched with the differential ion features between sample groups. Formula 2 is composed of glucose, isomaltose, and mannitol, three major compounds among the 28 validated metabolites. Formula 3 is composed of the other 25 minor compounds.

The mixture of chemical standards for all identified compounds (Formula 1 in Supplementary Data Sheet 6) achieved an astringency score of 38, significantly higher than the mean score of 33 in the N2&T group, but slightly lower than the mean score of 40 in group N1 (Figure 5). Additionally, Formula 1 showed a nasal moistening score of 32, matching that of the N2&T group, but slightly below the score observed in the N1 group. These results validated the effectiveness of the nontargeted metabolite analysis approach in identifying compound components associated with enhanced sensory attributes of astringency and nasal moistening among the investigated natural spices. We also developed Formula 2, comprising the three major components (glucose, isomaltose, and mannitol), and Formula 3, consisting of the remaining 25 ingredients at lower concentration levels (Supplementary Data Sheet 6). Interestingly, Formula 3 achieved an astringency score of 36, which closely approached the score of 38 for Formula 1. Moreover, its nasal moistening score was 34, slightly higher than the score of 32 assigned for Formula 1. In contrast, Formula 2 did not exhibit enhanced sensory attributes, with astringency and nasal moistening scores of 26 and 22, respectively. These findings suggest that the minor ingredients present at lower concentrations might play a more direct role in enhancing sensory evaluation scores compared to the major sugar components. This highlights the importance of nontargeted metabolite analysis in identifying minor chemical compounds that contribute to improved sensory performance, providing valuable insights for designing flavor additive formulas.

While nontargeted metabolite analysis was able to reveal a broad spectrum of chemical entities associated with improved sensory perceptions, validation using chemical standards identified only a small fraction of the extracted differential ion features (23 out of 1,250 for the N1 group and 5 out of 603 for the N2&T group). The primary limitation lay in the low coverage of natural products in existing mass spectral libraries, which hindered the successful annotation of most differential ion features. Additionally, although spectral matching may have suggested structural similarities between a detected ion feature and a recorded compound, large discrepancies in retention times (i.e., >0.5 min) reveal that the features are actually isomers of the annotated compounds. Furthermore, chemical standards for most annotated natural products were not commercially available. As a result, relying solely on compounds with confirmed molecular structures led to flavor additive formulas, such as Formula 1 and Formula 3, exhibiting slightly lower astringency and nasal moistening scores compared to the real samples clustered in the N1 group.

## 4 Conclusion

This study utilized a nontargeted metabolite analysis approach, using LC-HRMS and DIA-MS technologies, to identify metabolites linked to variations in sensory perceptions of 57 tobacco and natural spice extracts by smoking evaluation. These samples were evaluated and categorized based on their sensory evaluation scores using PCA analysis. Statistical analysis of the nontargeted datasets identified 1,853 differential ion features associated with enhanced astringency and nasal moistening attributes. Among these, 89 ion features were putatively annotated through mass spectral matching with library records, and 28 nonvolatile chemicals were further confirmed using available chemical standards. Noteworthy, the validation rate remained relatively low, primarily due to the limited coverage of mass spectral libraries for natural products and the scarcity of available chemical standards. Nevertheless, sensory evaluation of formulations containing the validated compounds demonstrated the effectiveness of nontargeted metabolite analysis in identifying key compounds that enhance sensory perceptions, even at low concentrations in the studied spices. While the limited number of validated compounds prevented us from fully reconstituting a formula equivalent to those in the N1 group, these preliminary findings demonstrated the utility of nontargeted metabolite analysis in revealing important flavor compounds. It is envisaged that the integration of nontargeted metabolite analysis with chemometrics could provide a promising digital approach for optimizing tobacco additive formulations.

## Data Availability

The datasets presented in this study can be found in online repositories. The names of the repository/repositories and accession number(s) can be found in the article/Supplementary Material.

## References

[B1] Baeshen N. A. Almulaiky Y. Q. Afifi M. Al-Farga A. Ali H. A. Baeshen N. N. (2023). GC-MS analysis of bioactive compounds extracted from plant rhazya stricta using various solvents. Plants 12 (4), 960. 10.3390/plants12040960 36840308 PMC9967519

[B2] Balbas J. Hamid N. Liu T. Kantono K. Robertson J. White W. L. (2015). Comparison of physicochemical characteristics, sensory properties and volatile composition between commercial and New Zealand made wakame from undaria pinnatifida. Food Chem. 186, 168–175. 10.1016/j.foodchem.2015.03.079 25976807

[B3] Banožić M. Jokić S. Ačkar Đ. Blažić M. Šubarić D. (2020). Carbohydrates—Key players in tobacco aroma formation and quality determination. Molecules 25 (7), 1734. 10.3390/molecules25071734 32283792 PMC7181196

[B4] Belov M. E. Anderson G. A. Angell N. H. Shen Y. Tolic N. Udseth H. R. (2001). Dynamic range expansion applied to mass spectrometry based on data-dependent selective ion ejection in capillary liquid chromatography fourier transform ion cyclotron resonance for enhanced proteome characterization. Anal. Chem. 73 (21), 5052–5060. 10.1021/ac010733h 11721899

[B5] Chen J. Li Y. He X. Jiao F. C. Xu M. L. Hu B. B. (2021). Influences of different curing methods on chemical compositions in different types of tobaccos. Ind. Crops Prod. 167, 113534. 10.1016/j.indcrop.2021.113534

[B6] Chen Y. Yang J. Meng Q. Tong H. (2023). Non-volatile metabolites profiling analysis reveals the tea flavor of “zijuan” in different tea plantations. Food Chem. 412, 135534. 10.1016/j.foodchem.2023.135534 36732104

[B7] Choudhury F. K. Pandey P. Meitei R. Cardona D. Gujar A. C. Shulaev V. (2022). GC-MS/MS profiling of plant metabolites. Methods Mol. Biol. 2396, 101–115. 10.1007/978-1-0716-1822-6_9 34786679

[B8] Collins B. C. Gillet L. C. Rosenberger G. Röst H. L. Vichalkovski A. Gstaiger M. (2013). Quantifying protein interaction dynamics by SWATH mass spectrometry: application to the 14-3-3 system. Nat. Methods 10(12)**,** 1246–1253. 10.1038/nmeth.2703 24162925

[B9] Cordell G. A. (2013). Fifty years of alkaloid biosynthesis in phytochemistry. Phytochemistry 91, 29–51. 10.1016/j.phytochem.2012.05.012 22721782

[B10] Cui K. Meng G. Liu Y. Qu Z. Ma J. Liu Q. (2015). Establishment and application of sensory evaluation method for cigarettes based on perceptual labeling. Tob. Sci. Technol. 48 (3), 5. 10.16135/j.issn1002-0861.20150313

[B11] Deshpande S. Matei M. F. Jaiswal R. Bassil B. S. Kortz U. Kuhnert N. (2016). Synthesis, structure, and tandem mass spectrometric characterization of the diastereomers of quinic acid. J. Agric. Food Chem. 64 (38), 7298–7306. 10.1021/acs.jafc.6b02472 27513177

[B12] Ding Y. Zhu L. J. Liu S. M. Yu H. Q. Dai Y. (2013). Analytical method of free and conjugated neutral aroma components in tobacco by solvent extraction coupled with comprehensive two-dimensional gas chromatography-time-of-flight mass spectrometry. J. Chromatogr. A 1280, 122–127. 10.1016/j.chroma.2013.01.028 23357748

[B13] Gigl M. Frank O. Barz J. Gabler A. Hegmanns C. Hofmann T. (2021). Identification and quantitation of reaction products from quinic acid, quinic acid lactone, and chlorogenic acid with strecker aldehydes in roasted coffee. J. Agric. Food Chem. 69 (3), 1027–1038. 10.1021/acs.jafc.0c06887 33433215

[B14] Graves B. M. Johnson T. J. Nishida R. T. Dias R. P. Savareear B. Harynuk J. J. (2020). Comprehensive characterization of mainstream marijuana and tobacco smoke. Sci. Rep. 10 (1), 7160. 10.1038/s41598-020-63120-6 32345986 PMC7188852

[B15] Guo J. Huan T. (2020). Comparison of full-scan, data-dependent, and data-independent acquisition modes in liquid chromatography–mass spectrometry based untargeted metabolomics. Anal. Chem. 92 (12), 8072–8080. 10.1021/acs.analchem.9b05135 32401506

[B16] Heuckeroth S. Damiani T. Smirnov A. Mokshyna O. Brungs C. Korf A. (2024). Reproducible mass spectrometry data processing and compound annotation in MZmine 3. Nat. Protoc. 19 (9), 2597–2641. 10.1038/s41596-024-00996-y 38769143

[B17] Hu W. Cai W. Li D. Liu Y. Luo C. Xue F. (2022). Exogenous additives facilitate the fermentation of cigar tobacco leaves: improving sensory quality and contents of aroma components. Food Sci. Technol. 42, e68122. 10.1590/fst.68122

[B18] Huang L.-F. Zhong K.-J. Sun X.-J. Wu M.-J. Huang K.-L. Liang Y.-Z. (2006). Comparative analysis of the volatile components in cut tobacco from different locations with gas chromatography-mass spectrometry (GC-MS) and combined chemometric methods. Anal. Chim. Acta 575 (2), 236–245. 10.1016/j.aca.2006.05.079 17723597

[B19] Jing B. Wang Z. Tan F. Guo Y. C. Tong S. R. Wang W. G. (2018). Hygroscopic behavior of atmospheric aerosols containing nitrate salts and water-soluble organic acids. Atmos. Chem. Phys. 18 (7), 5115–5127. 10.5194/acp-18-5115-2018

[B20] Li J. Ma Z. Dai H. Li H. Qiu J. Pang X. (2024). Application of PLSR in correlating sensory and chemical properties of middle flue-cured tobacco leaves with honey-sweet and burnt flavour. Heliyon 10 (8), e29547. 10.1016/j.heliyon.2024.e29547 38655300 PMC11035049

[B21] Liu B. Li Y. M. Wu S. B. Li Y. H. Deng S. S. Xia Z. L. (2013). Pyrolysis characteristic of tobacco stem studied by Py-GC/MS, TG-FTIR, and TG-MS. Bioresources 8 (1), 220–230. 10.15376/biores.8.1.220-230

[B22] Luo H. Cheng H. Du W. Wang S. Wang C. Chang S. (2013). Optimization extraction process of aroma components in tobacco. J. Chromatogr. Sci. 51 (3), 250–257. 10.1093/chromsci/bms136 22907910

[B23] Mitsui K. David F. Tienpont B. Sandra K. Ochiai N. Tamura H. (2015). Analysis of the reaction products from micro-vial pyrolysis of the mixture glucose/proline and of a tobacco leaf extract: search for amadori intermediates. J. Chromatogr. A 1422, 27–33. 10.1016/j.chroma.2015.10.021 26602543

[B24] Ni J.-B. Zielinska M. Wang J. Fang X.-M. Prakash Sutar P. Li S.-B. (2023). Post-harvest ripening affects drying behavior, antioxidant capacity and flavor release of peach via alteration of cell wall polysaccharides content and nanostructures, water distribution and status. Food Res. Int. 170, 113037. 10.1016/j.foodres.2023.113037 37316090

[B25] Perez R. A. Navarro T. de Lorenzo C. (2007). HS-SPME analysis of the volatile compounds from spices as a source of flavour in 'campo real' table olive preparations. Flavour Fragr. J. 22 (4), 265–273. 10.1002/ffj.1791

[B26] Rezk-Hanna M. Talhout R. Jordt S.-E. (2023). Sugars and sweeteners in tobacco and nicotine products: food and drug administration's regulatory implications. Nicotine Tob. Res. 25 (4), 838–840. 10.1093/ntr/ntac222 36148496 PMC10032193

[B27] Santana V. V. Martins M. A. F. Loureiro J. M. Ribeiro A. M. Rodrigues A. E. Nogueira I. B. R. (2021). Optimal fragrances formulation using a deep learning neural network architecture: a novel systematic approach. Comput. Chem. Eng. 150, 107344. 10.1016/j.compchemeng.2021.107344

[B28] Schrimpe-Rutledge A. C. Codreanu S. G. Sherrod S. D. McLean J. A. (2016). Untargeted metabolomics strategies-challenges and emerging directions. J. Am. Soc. Mass Spectrom. 27 (12), 1897–1905. 10.1007/s13361-016-1469-y 27624161 PMC5110944

[B29] Schumacher J. N. Green C. R. Best F. W. Newell M. P. (1977). Smoke composition. An extensive investigation of the water-soluble portion of cigarette smoke. J. Agric. Food Chem. 25 (2), 310–320. 10.1021/jf60210a003 838966

[B30] Schwanz T. G. Bokowski L. V. V. Marcelo M. C. A. Jandrey A. C. Dias J. C. Maximiano D. H. (2019). Analysis of chemosensory markers in cigarette smoke from different tobacco varieties by GC×GC-TOFMS and chemometrics. Talanta 202, 74–89. 10.1016/j.talanta.2019.04.060 31171230

[B31] Shiner L. (2015). Art scents: perfume, design and olfactory art. Br. J. Aesthet. 55 (3), 375–392. 10.1093/aesthj/ayv017

[B32] Song H. Liu J. (2018). GC-O-MS technique and its applications in food flavor analysis. Food Res. Int. 114, 187–198. 10.1016/j.foodres.2018.07.037 30361015

[B33] Sowalsky R. A. Noble A. C. (1998). Comparison of the effects of concentration, pH and anion species on astringency and sourness of organic acids. Chem. Senses 23 (3), 343–349. 10.1093/chemse/23.3.343 9669047

[B34] Stein S. E. Scott D. R. (1994). Optimization and testing of mass spectral library search algorithms for compound identification. J. Am. Soc. Mass Spectrom. 5 (9), 859–866. 10.1016/1044-0305(94)87009-8 24222034

[B35] Talhout R. Opperhuizen A. van Amsterdam J. G. C. (2006). Sugars as tobacco ingredient: effects on mainstream smoke composition. Food Chem. Toxicol. 44 (11), 1789–1798. 10.1016/j.fct.2006.06.016 16904804

[B36] Tsugawa H. Cajka T. Kind T. Ma Y. Higgins B. Ikeda K. (2015). MS-DIAL: data-independent MS/MS deconvolution for comprehensive metabolome analysis. Nat. Methods 12 (6), 523–526. 10.1038/nmeth.3393 25938372 PMC4449330

[B37] Vrzal T. Drábková K. Štěrba K. Olšovská J. (2021). Pilot sensomic study revealing the potential of amino acids to highly influence sensory properties of a lager beer. J. Food Compos. Anal. 102 **,** 104028. 10.1016/j.jfca.2021.104028

[B38] Wang Z. H. Li X. L. Zhen S. J. Li X. Y. Wang C. W. Wang Y. J. (2013). The important role of quinic acid in the formation of phenolic compounds from pyrolysis of chlorogenic acid. J. Therm. Anal. Calorim. 114 (3), 1231–1238. 10.1007/s10973-013-3142-z

[B39] Wang R.-Q. Ding J. Geng Y. Li Y.-Z. Mei Y.-W. Bao K. (2021). CRB-SWATH:a method for enhancing untargeted precursor ion extraction and automatically constructing their tandem mass spectra from swath datasets by chromatographic retention behaviors. Anal. Chem. 93 (36), 12273–12280. 10.1021/acs.analchem.1c01841 34459594

[B40] Wang R.-Q. Geng Y. Song J.-N. Yu H.-D. Bao K. Wang Y.-R. (2023). Biogenic solution map based on the definition of the metabolic correlation distance between 4-dimensional fingerprints. Anal. Chem. 95 (19), 7503–7511. 10.1021/acs.analchem.2c05480 37130068

[B41] Wang R.-Q. Wang Y. Wang M. Yu H.-D. Xu X.-J. Xu P. (2025). CRB-FCC: a standardized nontargeted analysis for formula assignment and structure annotation. Anal. Chem. 97 (42), 23204–23213. 10.1021/acs.analchem.5c03451 41111283

[B42] Wu L. J. He Z. J. Wu Y. S. Liu J. Y. Li C. Z. Cao J. L. (2013). Evaluation of aroma components in chinese southwest tobacco by headspace gas chromatography-mass spectrometry. Asian J. Chem. 25 (16), 8853–8858. 10.14233/ajchem.2013.14698

[B43] Wu X. Cai W. Zhu P. Peng Z. Zheng T. Li D. (2022). Profiling the role of microorganisms in quality improvement of the aged flue-cured tobacco. BMC Microbiol. 22 (1), 197. 10.1186/s12866-022-02597-9 35965316 PMC9377114

[B44] Yamaguchi N. Suzuki S. Makino A. (2013). Starch degradation by alpha-amylase in tobacco leaves during the curing process. Soil Sci. Plant Nutr. 59 (6), 904–911. 10.1080/00380768.2013.842884

[B45] Yeager S. E. Batali M. E. Guinard J.-X. Ristenpart W. D. (2023). Acids in coffee: a review of sensory measurements and meta-analysis of chemical composition. Crit. Rev. Food Sci. Nutr. 63 (8), 1010–1036. 10.1080/10408398.2021.1957767 34553656

[B46] Zhang X. Zhou T. Ng K. M. (2021). Optimization-based cosmetic formulation: integration of mechanistic model, surrogate model, and heuristics. AICHE J. 67 (1), e17064. 10.1002/aic.17064

[B47] Zhao L. Shang S. Tian Y. Gao Y. Song Z. Peng L. (2023). Integrative analysis of sensory evaluation and non-targeted metabolomics to unravel tobacco leaf metabolites associated with sensory quality of heated tobacco. Front. Plant Sci. 14, 1123100. 10.3389/fpls.2023.1123100 36844088 PMC9944805

[B48] Zhou B. Ma B. Xu C. Wang J. Wang Z. Huang Y. (2022). Impact of enzymatic fermentation on taste, chemical compositions and *in vitro* antioxidant activities in Chinese teas using E-tongue, HPLC and amino acid analyzer. LWT 163 **,** 113549. 10.1016/j.lwt.2022.113549

[B49] Zhu W. K. Wang Y. Chen L. Y. Wang Z. G. Li B. Wang B. (2016). Effect of two-stage dehydration on retention of characteristic flavor components of flue-cured tobacco in rotary dryer. Dry. Technol. 34 (13), 1621–1629. 10.1080/07373937.2016.1138965

[B50] Zou L. Su J. Xu T. Ji X. Wang T. Chen Y. (2023). Untargeted metabolomics revealing changes in aroma substances in flue-cured tobacco. Open Chem. 21 (1), 20220326. 10.1515/chem-2022-0326

