# Supplementary material for: Unraveling the molecular basis of sensory attributes in smoking spices: a nontargeted metabolite analysis using liquid chromatography high resolution mass spectrometry
Source: Front Mol Biosci. 2025 Dec 5;12:1687831. doi: 10.3389/fmolb.2025.1687831 (PMC12714608; doi:10.3389/fmolb.2025.1687831)
Supplement: Supplementary file 1 [file DataSheet2.pdf]

Appendix 1: Sensory Training Protocol for Nasal Moistening

A Sensometric Method for Assessing Nasal Moistening Sensation in Cigarette Smoke Using Sorbitol Calibration

1. Materials and Methods

1.1. Materials and Reagents

Base Cigarettes: A homogeneous batch of neutral, untreated cigarettes with consistent physical parameters and basic composition.

Chemical Calibrant: Food-grade D-Sorbitol.

Solvent: Ultra-pure water.

Syringe: A precision micro-syringe capable of accurate 1.0 μL deliveries.

1.2. Preparation of Calibrated Cigarettes

Sorbitol solutions were prepared in ultra-pure water at concentrations of 0%, 0.05%, 0.25%, 0.5%, 1%, and 2% (weight/volume).

A precise volume of 1.0 μL of each solution was injected into individual base cigarettes using the micro-syringe.

All prepared cigarettes were conditioned at (22 ± 1)°C and (60 ± 2)% relative humidity for a minimum of 24 hours to ensure stability before sensory analysis.

2. Establishment of the Sensory Reference Scale

Table 1 Standard and scores in reference of serial Sorbitol solutions

No.	Sorbitol Concentration (w/w)	Score	Sensory Descriptor
S0	0	1	Strongly dry: definite dryness, prickle, or burning sensation in the nasal cavity.
S1	0.05%	2	Distinctly dry: clear sensation of dryness, accompanied by slight irritation.
S2	0.25%	4	Slightly dry: perceivable dryness with only weak irritancy.
S3	0.5%	6	Neutral: No significant dryness or moisturizing sensation. Represents the acceptable baseline.
S4	1%	8	Distinctly moisturizing: clear sensation of moistness and smoothness; comfortable in the nasal cavity.
S5	2%	10	Strongly moisturizing: intense moisturizing and coating sensation; smoke is extremely smooth.

3. Panelist Training Procedure

3.1 Theoretical orientation

Panelists are introduced to the definition of " Nasal Moistening Sensation" as the physical sensation of moistness experienced when smoke is exhaled through the nose, which must be evaluated separately from olfactory aroma characteristics.

The concept of the sorbitol chemical scale and its correlation with the 10-point scoring system is explained.

3.2 Sequential Exposure and Scale Anchoring

Ordered exposure: Panelists smoke the calibrated cigarettes in a specific sequence: S0 - S5 - S1 - S2 - S3 - S4. This order establishes a robust sensory memory from the extreme dry anchor to the extreme moist anchor.

Lexicon alignment: Through guided discussion, panelists align their descriptive vocabulary with the standard descriptors provided in Table 1.

3.3 Blind Recognition and Ranking Calibration

The S0 to S5 cigarettes are presented in a randomized, blinded format.

Task 1 (identification): panelists are asked to identify samples corresponding to a specific concentration level (e.g., "Identify the S3 sample").

Task 2 (ranking): panelists must arrange all blinded samples in the correct order of increasing nasal moistening intensity.

Immediate feedback and group discussion are conducted to calibrate perceptions and resolve discrepancies.

3.4 Scoring Practice

Panelists practice scoring the known calibration samples against the 10-point reference scale from Table 1.

The focus is on consistency and accuracy in aligning individual scores with the predefined reference values.

4. Panelist Qualification and Formal Assessment

Qualification: To be certified, each panelist must evaluate one or two blinded validation samples (sorbitol-calibrated cigarettes with known concentrations).

Pass Criteria: The panelist's mean score for each validation sample must be within ± 0.5 points of the reference score, and they must correctly identify its concentration level (e.g., S2, S4).

Formal Assessment of Test Samples:

Prior to each session, qualified panelists re-smoke the S0 (Score 1) and S5 (Score 10) references to re-calibrate their sensory memory.

Test samples (e.g., cigarettes with natural flavorings) are evaluated in a randomized and blinded manner.

Panelists are instructed to disregard the aromatic character and focus solely on the physical sensation of moisturizing in the nasal passage.

Intensity scores are recorded based on the established sorbitol scale for subsequent statistical analysis.

This protocol provides a standardized and reproducible sensometric method for quantifying the nasal moistening properties of cigarette smoke, specifically tailored for the evaluation of natural flavorings.
